# How to Interpret Needle EMG Study: A Stepwise Approach

**DOI:** 10.1212/NE9.0000000000200370

**Published:** 2026-10-13

**Authors:** Gabriela Figueiredo Pucci, Jonathan Minkis, Marcus V. Pinto, Aaron S. Zelikovich

**Affiliations:** 1 Department of Neurology, University of Pittsburgh, PA;; 2 Northwell, New Hyde Park, NY; and; 3 Department of Neurology, Mayo Clinic, Rochester, MN.

Needle EMG is performed after nerve conduction studies (NCSs) to further localize various diseases from anterior horn cell to muscles. This Teaching Neurovisual is a visual aid to be used for teaching the interpretation of EMGs to medical students and neurology residents.

Step 1 is the evaluation of spontaneous activity (muscle at rest) (Figure 1). Step 2 is the study of the motor unit action potentials (muscle contracted). Step 3 involves identification of patterns and localization, with the conjunction with NCS (Figure 2). Step 4 is the identification of the time course to better characterize the abnormalities previously seen.^1,2^

**Figure 1:**
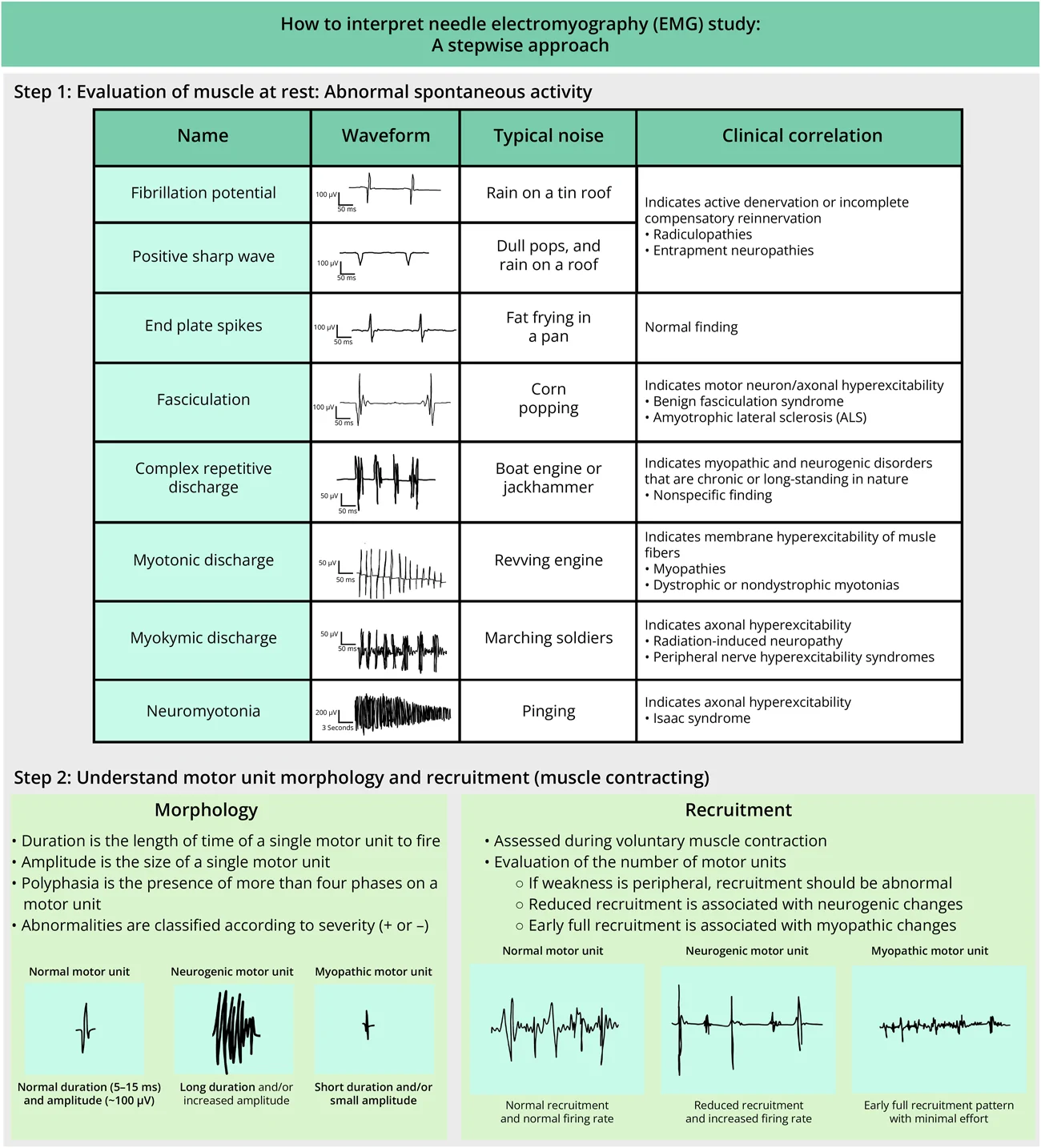
Evaluating Motor Units at Rest and With Activity In step 1, learners should evaluate muscles at rest and identify abnormal spontaneous activity patterns that may provide clues to the underlying pathology. In step 2, during muscle activation, learners should assess the characteristics of motor unit potentials, including their morphology and recruitment patterns.

**Figure 2:**
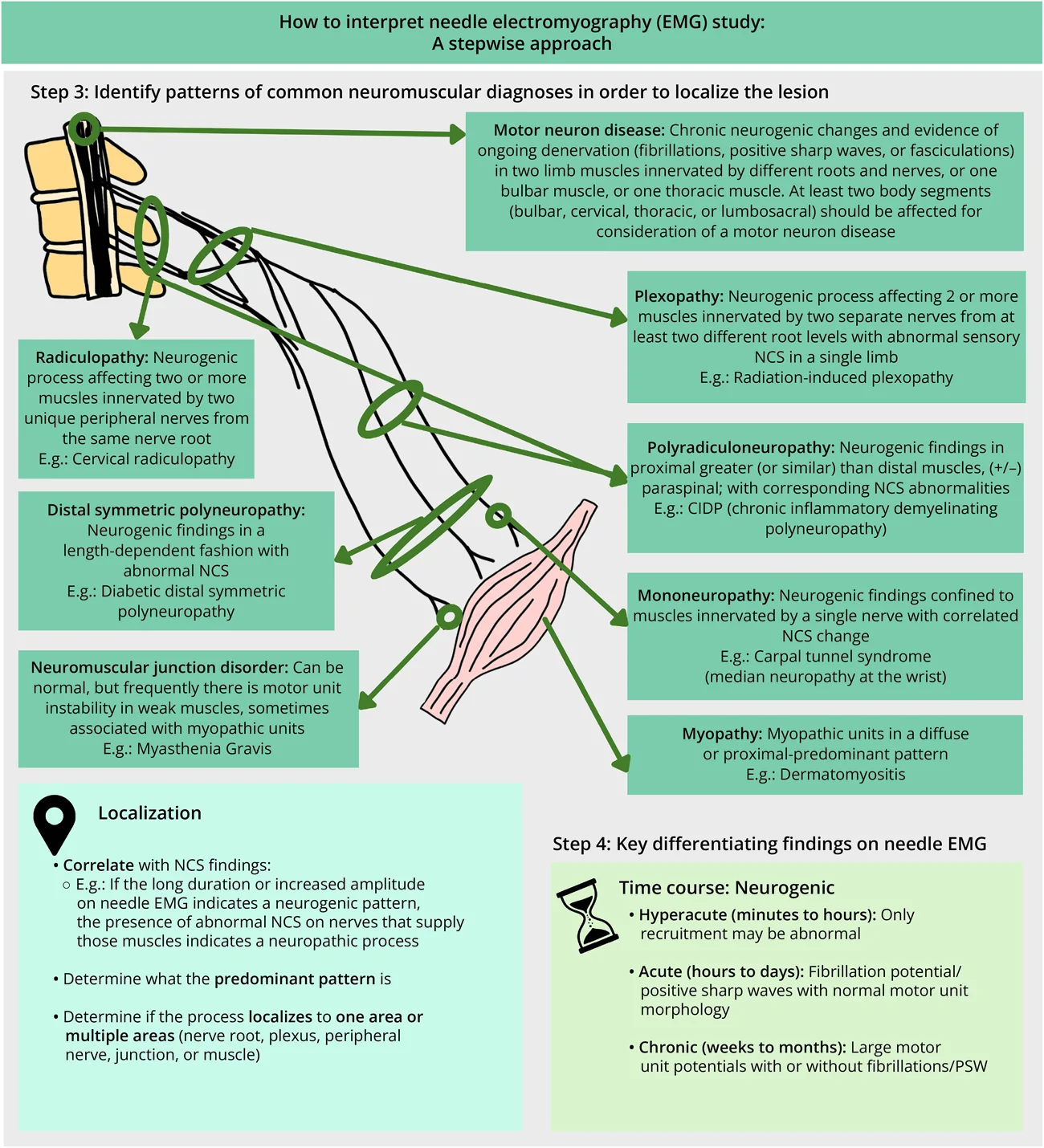
Interpreting EMG Findings in the Context of Clinical History and NCS In step 3, findings from steps 1 and 2 should be integrated to identify potential disease patterns. In step 4, electrodiagnostic findings should be correlated with the clinical history and results of nerve conduction studies. NCS = nerve conduction study.
